# A Fluorescent One-Dimensional Photonic Crystal for Label-Free Biosensing Based on Bloch Surface Waves

**DOI:** 10.3390/s130202011

**Published:** 2013-02-05

**Authors:** Francesca Frascella, Serena Ricciardi, Paola Rivolo, Valeria Moi, Fabrizio Giorgis, Emiliano Descrovi, Francesco Michelotti, Peter Munzert, Norbert Danz, Lucia Napione, Maria Alvaro, Federico Bussolino

**Affiliations:** 1 Dipartimento di Scienza Applicata e Tecnologia, Politecnico di Torino, corso Duca degli Abruzzi 24, Torino 10129, Italy; E-Mails: francesca.frascella@polito.it (F.F.); serena.ricciardi@polito.it (S.R.); paola.rivolo@polito.it (P.R.); valeria.moi@polito.it (V.M.); fabrizio.giorgis@polito.it (F.G.); 2 Dipartimento di Scienze di Base e Applicate per l'Ingegneria, SAPIENZA Università di Roma, via A. Scarpa 16, Roma 00161, Italy; E-Mail: francesco.michelotti@uniroma1.it; 3 Fraunhofer Institute for Applied Optics and Precision Engineering, Albert-Einstein-Str. 7, Jena 07745, Germany; E-Mails: peter.munzert@iof.fraunhofer.de (P.M.); norbert.danz@iof.fraunhofer.de (N.D.); 4 Dipartimento di Scienze Oncologiche, Istituto per la Ricerca e la Cura del Cancro, Università di Torino, Candiolo 10060, Italy; E-Mails: lucia.napione@ircc.it (L.N.); maria.alvaro@ircc.it (M.A.); federico.bussolino@ircc.it (F.B.)

**Keywords:** surface electromagnetic waves, label-free sensing, fluorescence, multilayers, bragg mirrors

## Abstract

A one-dimensional photonic crystal (1DPC) based on a planar stack of dielectric layers is used as an optical transducer for biosensing, upon the coupling of TE-polarized Bloch Surface Waves (BSW). The structure is tailored with a polymeric layer providing a chemical functionality facilitating the covalent binding of orienting proteins needed for a subsequent grafting of antibodies in an immunoassay detection scheme. The polymeric layer is impregnated with Cy3 dye, in such a way that the photonic structure can exhibit an emissive behavior. The BSW-coupled fluorescence shift is used as a means for detecting refractive index variations occurring at the 1DPC surface, according to a label-free concept. The proposed working principle is successfully demonstrated in real-time tracking of protein G covalent binding on the 1DPC surface within a fluidic cell.

## 1. Introduction

During the last years, many different fields of research like biology, biochemistry and pharmaceutics share a need of employing new sensing techniques that allow the detection of small quantities of analyte in a liquid or gaseous samples. Surface plasmon resonance (SPR) sensing [1] is an optical label-free technique that can be easily used for this purpose: it shows high sensitivity, good reproducibility and selectivity, and commercial SPR platforms are now available [2]. SPR and plasmon-related techniques rely essentially on the exploitation of electromagnetic fields strongly confined on the surface of metallic films.

In alternative to Surface Plasmon Polariton (SPP) waves, surface modes on photonic crystals can be used instead. As an example, periodic multilayered structures (or one-dimensional photonic crystals-1DPC) represent a promising platform for implementing sensing schemes based on the coupling of Bloch Surface Waves (BSW) [3,4]. Although photonic structures with higher dimensionalities can be used to sustain surface modes [5], the BSWs we consider here can be either TE- or TM- polarized electromagnetic waves propagating at the surface of properly designed dielectric 1DPC [6–8].

The use of BSWs as an optical transducer presents some advantages, such as spectral and polarization tunability and low losses. The resulting sharp resonances associated to coupled BSWs, e.g., according to the Kretschmann configuration, can improve the figure-of-merit of the sensing performances as compared to SPR [9]. Thanks to the available technologies, periodic stacks of layers having different refractive indices can be obtained, wherein BSW can be coupled in a broad spectral range, from the near-infrared [10] to the visible [11,12], with TE and TM polarizations [13].

Recently, surface modes on 1DPC have been used for demonstrating label-free detection schemes based on enhanced diffraction [14,15], spectral/angular resonance shift [16–20], or to improve fluorescence-based detection [21–23]. To this extent, 1DPC sustaining BSWs represent a powerful platform combining most of the sensing possibilities offered by conventional SPR and photonic-crystal based fluorescence detection [24–26].

This work describes a label-free biosensing technique based on BSW. When dealing with low-losses 1DPCs, a limiting factor can be represented by the shallowness of the BSW resonance dip. This aspect might negatively affect the detection range of a refractometric measurement because of the small contrast of the resonance as detected in the far field. Here, we show an alternative approach to the standard reflectance-based setup, overcoming the limitation above. In fact, we present a refractometric scheme implemented by monitoring the shift of a BSW-coupled luminescence peak radiated from a proper (fluorescent) 1DPC in which BSWs are resonantly laser-excited [27]. BSWs are coupled by prism-illuminating the multilayer with a laser light (symbol = 532 nm) incident at a given angle in accordance to the BSW dispersion curve. The BSW dispersion curve depends on the materials and the geometrical layout of the multilayer. Laser-coupled BSW produces a strong near-field enhancement at the multilayer surface, useful for boosting the fluorescence emission of organic dyes suitably placed on the multilayer top. Like the well known Surface Plasmon Coupled Emission (SPCE), the radiated fluorescence couples into the BSW and leaks out of the prism with a leakage angle according to the BSW dispersion curve. When a refractive index perturbation occurs close to the multilayer surface, the BSW dispersion shifts (both angularly and spectrally). In our particular implementation, such a shift is spectrally tracked by detecting the BSW-coupled fluorescence leaking out of the prism with a fixed angle.

Accordingly, the proposed technique can be defined as label-free since the emitted radiation concerns with the photonic structure itself rather than a specifically labeled analyte. By exploiting the well-known effect of fluorescence coupling into available photonic/plasmonic modes [28], very narrow BSW-coupled peaks can be obtained with a better contrast as compared to the corresponding dips obtained by standard reflectance measurements.

One further advantage of using 1DPC as a platform for coupling surface modes is represented by the possibility to include in the multilayer design any additional functional layer required for grafting/immobilizing molecular receptors or probes suited for the specific biosensing. In the following, we demonstrate how a thin plasma-deposited polymeric film (Poly Acrylic Acid) impregnated by a Cy3 solution can be used to: (i) properly tailor an underlying dielectric multilayer in such a way that BSWs can be coupled; (ii) provide a distributed, fluorescence source; (iii) provide a COOH-rich substrate suitable to promote protein G immobilization, through the formation of amidic bonds.

## 2. Experimental Section

### 2.1. Photonic Structure

**Figure 1.**
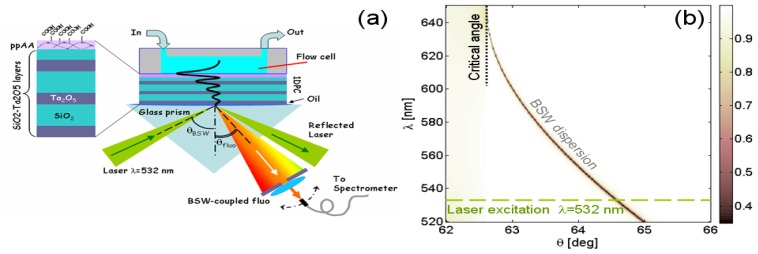
(**a**) Sketch of the photonic structure and the experimental configuration for BSW-controlled fluorescence. (**b**) Calculated reflectivity map R (θ, λ) of the 1DPC in water environment.

The 1DPC is based on six layers of alternating low (L) and high (H) index materials (SiO_2_ and Ta_2_O_5_, respectively) deposited by plasma ion assisted deposition under high vacuum conditions, using an APS904 coating system (Leybold Optics) on a glass substrate Figure 1(a). The thickness of each layer is specified by the following stack sequence glass–δ_H_–δ_Λ_–δ_H_–δ_Λ_–δ_H_–δ_Λ_′–air, where δ_H_ = 105 nm, δ_Λ_ = 315 nm and δ_Λ_′ = 125 nm. On top of this stack, a δ_Π_ = 100 nm polyacrylic Acid (PPAA) is plasma-deposited. The structure is able to sustain TE-polarized BSW in the visible range, when the outer medium contacting the last (PPAA) layer is an aqueous solution.

In order to make the 1DPC inherently fluorescent, the last PPAA layer has been impregnated with a 1 mL ethanolic solution of Cy3 dye at a concentration of 10^−5^ M. Fluorescence is then excited upon laser coupling of a BSW (λ = 532 nm) in the Kretschmann configuration and then collected at a specific leakage angle by means of a fibered spectrometer (Ocean Optics USB2000+). The illumination beam is slightly focused (NA = 0.1). In Figure 1(b), a calculated reflectivity map R (λ, θ) of the 1DPC in the Kretschmann configuration is shown, wherein the narrow low-reflectivity region indicates the dispersion curve for BSWs. According to this designed dispersion curve, laser BSW can be coupled at an internal coupling angle θ_BSW_ = 64.7 deg and the BSW-coupled fluorescence can be collected at internal angles roughly ranging from 62.5 deg to 64.5 deg.

### 2.2. Polymeric Film

Plasma-Polymerized Acrylic Acid (PPAA) thin functional films are obtained in a 13.56 MHz Radio Frequency-Plasma Enhanced Chemical Vapour Deposition (RF-PECVD) reactor by means of a pulsed plasma discharge. This coating exposes at the surface a high number of carboxylic groups (−COOH) able to react with the amino groups (−NH_2_) of biomolecules.

During deposition, argon (Ar) is used as a gas carrier (flow = 20 sccm). In order to reach a thickness of 100 nm, the following parameters are selected: (a) D.C.% = 10; t_on_ = 10 ms; t_off_ = 90 ms; (b) Power_RF_ = 200 W; (c) deposition time 15 min. Before deposition an Ar etching step has been performed, in order to clean and activate the surface [29].

The physical-chemical properties of PPAA films deposited on several substrates are characterized by means of ATR FT-IR Spectroscopy, Optical Contact Angle (OCA). The surface density of the carboxylic functionalities is quantified by colorimetric titration with Toluidine Blue O (TBO).

Surface properties such as wettability and hydrophilicity of PPAA films have been investigated by OCA measurements on a OCAH 200 equipment (Dataphysics Instruments GmbH). Deionized water MilliQ grade (H_2_O) and diiodomethane (CH_2_I_2_-Sigma Aldrich) are used as liquids (droplet volume = 1.5 μL), for the analysis using the sessile droplet method in static mode. Droplet profiles have been fitted according to the Young-Laplace method. Surface free energy of bare and PPAA-coated substrates (PE, Corning glass, silicon) are determined using the Owens-Wendt-Kaelble (OWK) method.

In Table 1 the values of water and diiodomethane contact angles (OCA_H_2_O_ and OCA_CH_2_I_2__) measured on PPAA film after ddH_2_O rinsing (30 min) and drying under N_2_ flux are reported. OCA values measured on the corresponding PPAA coating deposited on different substrates demonstrate that the film surface properties do not depend on the nature of the substrate, providing a comparable roughness.

ATR FT-IR spectra are acquired on a Nicolet 5,700 FTIR Spectrometer (ThermoFisher) equipped with a DTGS detector. Spectroscopic resolution is 4 cm^−1^ and 64 interferograms are collected for each spectrum. The surface of PPAA films, put in close contact with a diamond crystal, are analysed in Attenuated Total Reflection (ATR) mode.

PE and Corning glass are considered as substrates. These two materials give rise to detectable IR absorptions because the penetration depth of the produced evanescent wave is larger than the thickness of typical deposited PPAA film. Therefore, substrate contributions hide PPAA features in different complementary portions of the selected spectral range.

**Figure 2.**
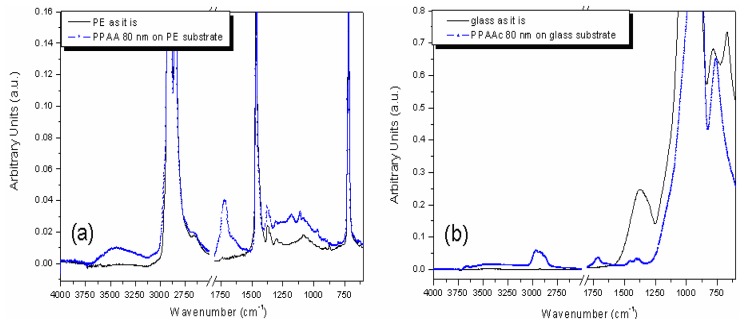
(**a**) ATR FT-IR spectra measured on bare PE and PPAA-PE substrate after ddH_2_O 30 min. rinsing. (**b**) ATR FT-IR spectra measured on bare glass and PPAA-glass substrate after ddH_2_O 30 min. rinsing.

The graph in Figure 2(a) shows the broad band at 3,250 cm^−1^ assigned to interacting −OH groups. Such a vibrational fingerprint, corroborated by the OCA characterizations, shows that a high amount of −COOH groups interconnected through aliphatic chains is obtained on the film surface. The peak at 1,730–1,710 cm^−1^ refers to the absorption of C=O stretching, corresponding to both carboxyl and carbonyl functionality. Moreover, at 1,200 cm^−1^ the C–O stretching mode is observed.

The graph in Figure 2(b) show the signals at 2,980–2,870 cm^−1^ related to aliphatic C–H stretching mode and as for PE substrate at 1,730–1,710 cm^−1^ the absorption related to C=O stretching. Finally, the C–H bending mode at 1,460–1,370 cm^−1^. Finally, in order to quantitatively estimate the concentration of the −COOH groups available at the film surface for a covalent binding with protein G a colorimetric titration with Toluidine Blue O (TBO, Sigma-Technical Grade) has been performed on PPAA film yielding a density value of ∼10^16^ −COOH/cm^2^.

## 3. Results and Discussion

### 3.1. Sensitivity Estimation

**Figure 3.**
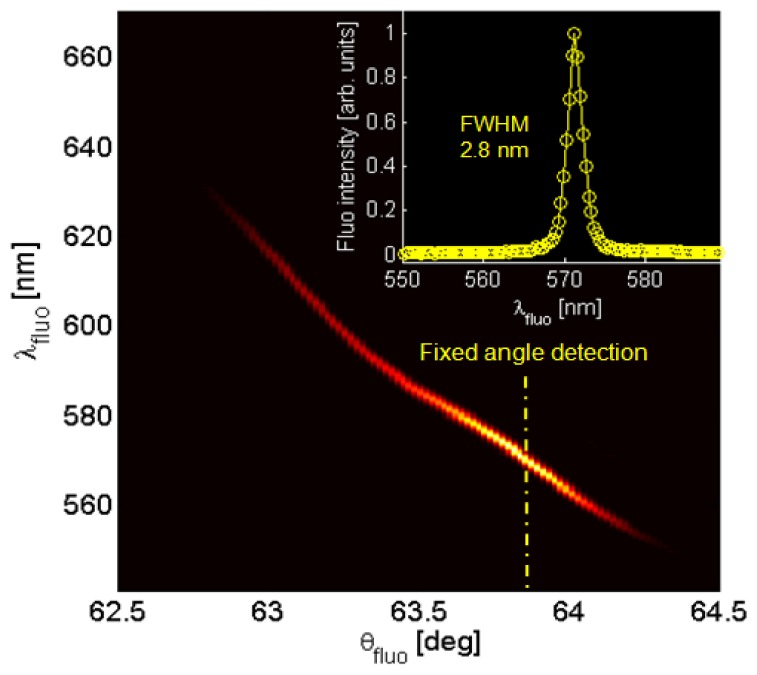
Experimentally measured angularly-resolved BSW-controlled fluorescence spectrum. Typical spectral widths at a given collection angle are below 3 nm.

According to the goniometric spectroscopic scheme as depicted in Figure 1(a), angularly resolved fluorescence spectra are collected. The 1DPC is contacted with water and the laser excitation is resonantly coupled to BSW, in order to take advantage of the near field enhancement as reported elsewhere [21]. After blocking the reflected laser beam, a typical luminescence map F(θ_fluo_, λ_fluo_) is obtained as shown in Figure 3, in which the illumination is kept fixed at the BSW coupling angle for λ = 532 nm whilst the collection is angularly scanned in the range 62.5 deg < θ_fluo_ < 64.5 deg. Laser light is blocked by a diaphragm and an edge filter (Semrock 532 nm RazorEdge^®^ ultrasteep long-pass edge filter). The detected fluorescence is mainly TE-polarized and shows an angular/spectral dispersion according to the BSW dispersion. For each collection angle θ_fluo_ a narrow BSW-coupled fluorescence peak is obtained, having a spectral width smaller than 3 nm (Figure 3, inset). The determined spectral width is proportional to the natural BSW resonance width convolved to the angular acceptance of the collection optics and it is not limited by the spectrometer resolution (<0.5 nm). The bright fluorescence peaks that can be obtained in this way result from the enhanced excitation provided by the BSW-coupled laser illumination and the coupled emission into BSW modes [21].

**Figure 4.**
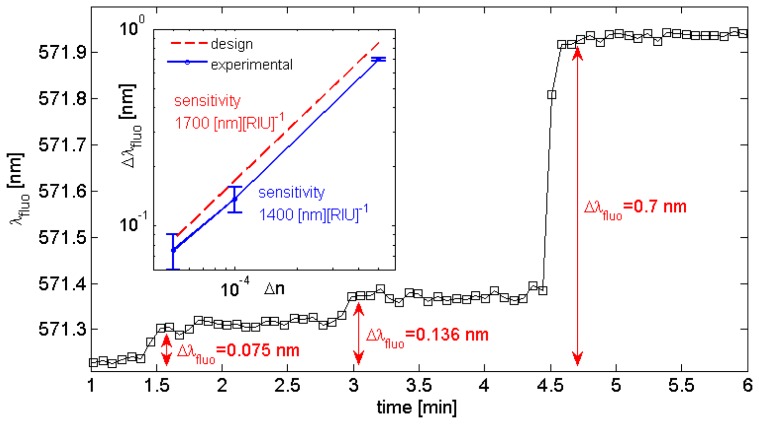
Spectral shift of BSW-coupled fluorescence upon injection of glucose solutions at different concentrations for the estimation of the sensitivity Δλ_fluo_/Δn (Δn = 5e^−5^; 1e^−4^; 5e^−4^). Inset: 1DPC sensitivity as experimentally estimated (blue solid line) and predicted from calculated design (red dashed line).

Prior to a real biosensing experiment, an estimate of the sensitivity of this optical transducer is needed. To this aim, progressive increases of the water refractive index are induced by subsequent injections of glucose solutions into the microfluidic flow cell at different concentrations. This procedure has been successfully used in the past [27]. Given the well known relationship between the glucose concentration *C* [mg][dL]^−1^ and the corresponding refractive index change Δn with respect to pure water [Δn(*C*) = α*C*, where α = 1.514e^−6^ [dL] [mg]^−1^ [30]], we could perturb the aqueous medium according to Δn = (5e−5, 1e−4, 5e−4). In Figure 4, the real-time spectral position of the BSW-coupled fluorescence is plotted as the water solution is fed with glucose solutions at increasing concentrations. The initial position is λ_fluo_ = 571.2 nm, corresponding to a detection angle kept fixed at θ_fluo_ ∼63.8 deg.

The overall spectral shift produced by the change of the refractive index in the aqueous medium is estimated after few seconds, when the signal is roughly stabilized. Then, an estimation of the noise is performed by taking into account the in-time fluctuations of the peak position about a mean value. When the estimated spectral shift values Δλ_fluo_ are plotted against the corresponding Δn (Figure 4, inset) an estimation of the sensitivity Δλ_fluo_/Δn ∼1,400 nm/RIU is obtained. Typically, the uncertainty level is affected by the BSW-coupled fluorescence intensity and it has been estimated as δΔλ_fluo_ = 3 × 10^−2^ nm in the present case. In Figure 4 (inset), the expected sensitivity from the calculated design is also reported.

### 3.2. Covalent Binding of Protein G

**Figure 5.**
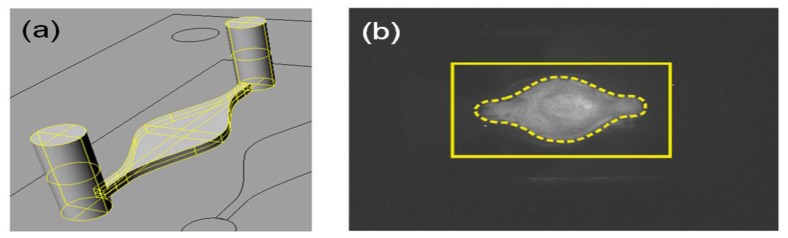
(**a**) Drawing of the flow cell used for protein incubation (volume 18.6 μL, Area = 0.744 cm^2^, height = 250 μm). (**b**) Chemiluminescence picture showing protein G binding on PPAA coated corning glass (yellow contour). The area exposed to the flow solution containing HRP-conjugated protein G (15 μg) is indicated by the yellow dashed contour.

To set up the experimental conditions for the absolute amount of protein to be used, we designed a chemiluminescence-based assay combining the use of horseradish peroxidase (HRP)-conjugated protein G (Thermo Scientific) with a chemiluminescence detection system suitable to assess the protein-surface binding on the total PPAA-coated surface area. Briefly, PPAA coated corning glasses were assembled in the fluidic cell system and exposed to different absolute quantities of HRP-conjugated protein G (150–15 μg). After washing cycles in PBS to remove protein excess, PPAA coated glasses were disassembled and exposed to enhanced chemiluminescence substrate (ECL; Perkin Elmer) producing a chemiluminescent signal as a result of ECL substrate-HRP reaction. The chemiluminescent signal was detected and recorded using ChemiDoc XRS charge-coupled device camera and Quantity One software (Bio-Rad). The Chemiluminescent signal indicates the presence of protein G binding onto the surface of a PPAA coated glass. As shown in Figure 5, an absolute quantity of 15 μg of HRP-protein G flowing in a fluidic circuit with an overall volume ∼250 μL (pump + tubing + flow cell), is sufficient to provide a homogeneous distribution of protein-surface binding.

In order to check the sensing capabilities of the BSW-based transducer, the covalent binding of protein G has been monitored in time. The formation of a homogeneous and saturated coverage of the sensor surface with protein G is an essential step for the subsequent immobilization of functional antibody receptors [31,32]. Although the selected model possesses little biological interest by itself, it represent nevertheless a simple and clean test because the covalent binding is irreversible and the experiment can be carried out neglecting dissociation phenomena.

To this aim, the 1DPC descried above has been contacted with the flow cell filled with an initial PBS solution. The solution is circulated by means of a piezo-micropump connected to the cell via flexible tubing. The pump can be controlled in such a way that a constant flow rate of ∼200 μL·min^−1^ is produced inside the fluidic circuitry. The sample solution (0.43 μg·μL^−1^) contains 130 μg of protein G (*i.e.*, an overall amount slightly less than 10 times larger than lower limit estimated to achieve a homogenous coating of the 1DPC surface within the flow cell) diluted in PBS (300 μL).

**Figure 6.**
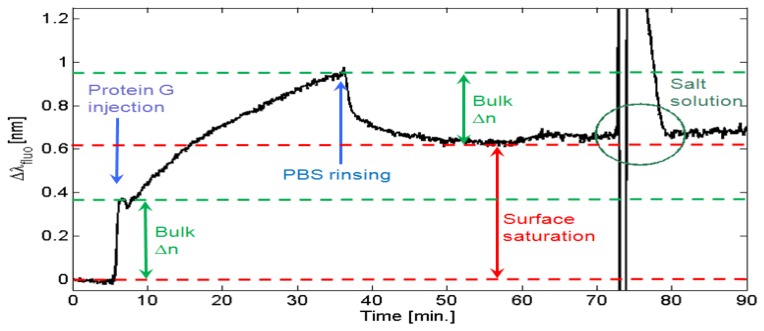
Real-time sensorgram showing the covalent binding of protein G onto the functional polymeric film during incubation in PBS buffer.

In Figure 6, an illustrative sensorgram is presented, showing how the protein G is progressively binding to the −COOH groups of the PPAA surface till an almost saturated coverage is reached. The plot provides the BSW peak shift Δλ_fluo_ as a function of time.

Initially, the BSW peak position is left stabilizing in a PBS buffer solution. After few minutes, a 300 μL volume of the protein G sample solution in injected and then made recirculating. At injection, a fast redshift of the BSW peak is detected. Subsequently, the redshift monotonically continues with a typical trend indicating the approach of an asymptotic condition. During this stage, protein G molecules are grafting onto the polymeric surface. After washing and recirculating with PBS solution, the BSW peak experiences an initial quick blueshift that stabilizes after few minutes at a Δλ_fluo_ = 0.6 nm with respect to the initial position. This overall redshift is due to the surface coverage of protein G. Interestingly, the blueshift observed during washing is quite comparable to the redshift observed at the injection of protein G. We account those spectral shifts mainly to the bulk refractive index changes of the solution only (indicated as “Bulk Δn” in Figure 6). In order to ensure that no protein G overlayers (*i.e.*, excess of protein G molecules eventually stitched on the surface due to electrostatic forces and not by virtue of the amidic bond), the flow rate has been increased at t = 60 min. This effect is perturbing the measurement of the BSW peak. However, no substantial blueshift due to the removal of an eventual excess of protein G is produced. A more effective removal of eventual overlayers of protein G is performed by introducing a highly concentrated NaCl solution [33]. A 1 M NaCl solution is injected at t = 73 min and the resulting refractive index perturbation is so strong that the BSW peak runs out of range. After washing with PBS, the BSW peak is substantially recovered, meaning that no overlayers are present. However, some additional redshift is observed. We account this effect to an excess of protein G previously uptaken by the fluidic circuit walls and then removed by the NaCl solution and re-diluted in the final PBS solution.

A condition of surface saturation is then observed by performing a second injection of the protein G solution. The resulting sensorgram (not shown) shows a redshift at injection and an opposite blueshift about the same amount at washing, therefore indicating that no a significant additional protein G can be immobilized on the PPAA surface.

## 4. Conclusions

In this work, BSW-controlled fluorescence is presented as a refractometric means for real-time tracking the covalent binding of a protein on a COOH-rich polymeric film. The photonic crystal structure employed to this aim is constituted by a dielectric multilayer tailored by a thin PPAA film. The PPAA film possess a twofold feature: it exhibits a “photonic” functionality for making BSWs appearing, and a “chemical” functionality for exhibiting COOH groups useful for molecule grafting. In addition, the polymeric layer has been made intrinsically fluorescent upon impregnation of a Cy3 dye. Fluorescence emitted by the dye is coupled at each wavelength to a BSW and collected under the corresponding BSW coupling angle. The result is a very narrow peak whose spectral shift can be monitored upon refractive index perturbations. The covalent binding of protein G in PBS buffer solution is time-monitored for providing a simple proof-of-principle of the detection scheme. Due to the irreversible and stable nature of the covalent binding, no dissociation takes place at the PPAA surface. It is therefore rather simple to get insights about the surface saturation by protein G and the formation of eventual protein G overlayers without being strongly affected by diffusion phenomena.

The proposed scheme overcomes the limitation in the signal-to-noise ratio occurring in standard angularly-resolved ATR reflectometry when low-losses materials are used for the photonic crystal. Furthermore, it opens new perspectives in using photonic crystal modes to tailor the angular radiation patterns of dyes located in close proximity of the sensing surface.

## Figures and Tables

**Table 1.** Contact angle characterization showing the OCA_H_2_O_ and OCA_CH_2_I_2__ values on different substrates coated with PPAA films and corresponding calculated surface energies (SE) (polar and dispersive contributions).

**Substrate**	**OCA_H_2_O_** **[deg ]**	**OCA_CH_2_I_2__** **[deg]**	**SE** **[mN·m^−1^]**	**SE disp** **[mN·m^−1^]**	**SE pol** **[mN·m^−1^]**
PE	90 ± 3	49 ± 3	35	33	2
PPAA on PE	62 ± 1	32 ± 1	48	36	12
Glass	42 ± 5	41 ± 2	57	26	31
PPAA on glass	59 ± 2	32 ± 3	50	36	14
Si	84 ± 1	31 ± 3	44	42	2
PPAA on Si	56 ± 3	31 ± 0	52	37	15
